# {4-Chloro-2-[(2-hy­droxy­eth­yl)imino­meth­yl]phenolato}{4-chloro-2-[(2-oxido­eth­yl)imino­meth­yl]phenolato}cobalt(III)

**DOI:** 10.1107/S1600536810033088

**Published:** 2010-08-21

**Authors:** Li-Jun Liu

**Affiliations:** a Experimental Center, Linyi Normal University, Linyi Shandong 276005, People’s Republic of China

## Abstract

In the title mononuclear cobalt(III) compound, [Co(C_9_H_8_ClNO_2_)(C_9_H_9_ClNO_2_)], the Co^II^ atom is six-coordinated by two imine N atoms, two phenolate O atoms, and one hy­droxy and one oxide O atom from two Schiff base ligands, forming an octa­hedral geometry. In the crystal structure, adjacent mol­ecules are linked through inter­molecular O—H⋯O hydrogen bonds. The 2-oxidoethyl group is disordered over two positions in a 0.638 (3):0.362 (3) ratio.

## Related literature

For general background to Schiff base cobalt(III) complexes, see: Zhang *et al.* (2010 ▶); Rodriguez *et al.* (2010 ▶); Khalaji *et al.* (2010 ▶); Luo & Luo (2010 ▶). For related cobalt complexes with octa­hedral coordination, see: De *et al.* (2001 ▶); Sun (2005 ▶); Zhu *et al.* (2003 ▶); Yuan (2006 ▶).
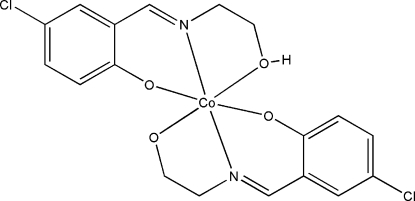


## Experimental

### 

#### Crystal data

[Co(C_9_H_8_ClNO_2_)(C_9_H_9_ClNO_2_)]
*M* *_r_* = 455.17
Hexagonal, 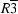

*a* = 18.675 (2) Å
*c* = 27.595 (3) Å
*V* = 8334.6 (16) Å^3^
*Z* = 18
Mo *K*α radiation
μ = 1.24 mm^−1^
*T* = 298 K
0.32 × 0.30 × 0.27 mm

#### Data collection

Bruker APEXII CCD area-detector diffractometer
Absorption correction: multi-scan (*SADABS*; Sheldrick, 2004 ▶) *T*_min_ = 0.692, *T*_max_ = 0.730
13818 measured reflections
4045 independent reflections
2390 reflections with *I* > 2σ(*I*)
*R*_int_ = 0.126

#### Refinement

*R*[*F* ^2^ > 2σ(*F* ^2^)] = 0.049
*wR*(*F* ^2^) = 0.135
*S* = 1.00
4045 reflections
257 parameters
9 restraints
H atoms treated by a mixture of independent and constrained refinement
Δρ_max_ = 0.40 e Å^−3^
Δρ_min_ = −0.44 e Å^−3^

### 

Data collection: *APEX2* (Bruker, 2004 ▶); cell refinement: *SAINT* (Bruker, 2004 ▶); data reduction: *SAINT*; program(s) used to solve structure: *SHELXS97* (Sheldrick, 2008 ▶); program(s) used to refine structure: *SHELXL97* (Sheldrick, 2008 ▶); molecular graphics: *SHELXTL* (Sheldrick, 2008 ▶); software used to prepare material for publication: *SHELXTL*.

## Supplementary Material

Crystal structure: contains datablocks global, I. DOI: 10.1107/S1600536810033088/hg2699sup1.cif

Structure factors: contains datablocks I. DOI: 10.1107/S1600536810033088/hg2699Isup2.hkl

Additional supplementary materials:  crystallographic information; 3D view; checkCIF report

## Figures and Tables

**Table 1:** Selected bond lengths (Å)

Co1—O1	1.874 (2)
Co1—O3	1.877 (3)
Co1—N1	1.887 (3)
Co1—N2	1.897 (3)
Co1—O4	1.916 (3)
Co1—O2	1.918 (3)

**Table 2:** Hydrogen-bond geometry (Å, °)

*D*—H⋯*A*	*D*—H	H⋯*A*	*D*⋯*A*	*D*—H⋯*A*
O2—H2⋯O4^i^	0.86 (1)	1.59 (1)	2.436 (4)	173 (5)

Symmetry code: (i) 
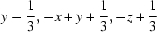
.

**Fig. 1.**
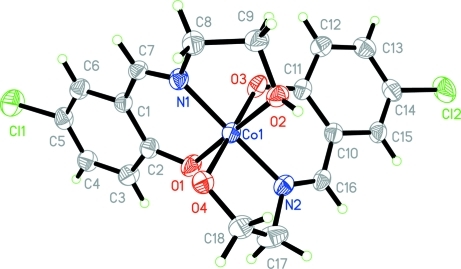
The molecular structure of the title complex with 30% probability displacement ellipsoids.

**Fig. 2.**
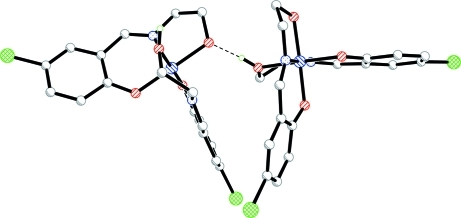
The crystal packing of the title complex, viewed along the c axis. Hydrogen bonds are shown as dashed lines.

## supplementary crystallographic information

### Comment

Cobalt(III) complexes with Schiff bases have been widely investigated in coordination chemistry and biological chemistry (Zhang *et al.*, 2010; Rodriguez *et al.*, 2010; Khalaji *et al.*, 2010; Luo & Luo, 2010). In the present paper, the title new cobalt(III) complex with the Schiff base ligand 4-chloro-2-[(2-hydroxyethylimino)methyl]phenol, is reported.

The Co^III^ atom in the title complex (Fig. 1) is six-coordinated by two imine N atoms, two phenolate O atoms, and two hydroxy O atoms from two Schiff base ligands, forming an octahedral geometry. The mainly difference in the two ligands is that one of the hydroxy groups is deprotonated. The bond lengths and angles (Table 1) related to the Co atom are comparable with those observed in similar cobalt complexes with octahedral geometry (De *et al.*, 2001; Sun, 2005; Zhu *et al.*, 2003; Yuan, 2006). In the crystal structure, the adjacent molecules are linked through intermolecular O—H···O hydrogen bonds (Table 2, Fig. 2).

### Experimental

5-Chlorosalicylaldehyde (0.1 mmol, 15.6 mg), 2-(2-aminoethylamino)ethanol (0.1 mmol, 10.4 mg), and cobalt acetate tetrahydrate (0.1 mmol, 24.9 mg) were mixed and stirred in methanol (20 ml) at reflux for 2 h, to give a red solution. The solution was cooled to room temperature, and red block-shaped single crystals were formed by slow evaporation of the solution in air. The characteristic IR absorption for the hydroxy group is at 3327 cm^-1^.

### Refinement

Atom H2 attached to O2 was located in a difference Fourier map and refined isotropically, with the O–H distance restrained to 0.85 (1) Å. The remaining H atoms were positioned geometrically (C–H = 0.93–0.97 Å) and refined using a riding model, with *U*_iso_(H) = 1.2*U*_eq_(C). The C8 atom is disordered over two distinct sites, with occupancies of 0.638 (3) and 0.362 (3).

### Crystal data

**Table:**

[Co(C_9_H_8_ClNO_2_)(C_9_H_9_ClNO_2_)]	*D*_x_ = 1.632 Mg m^−^^3^
*M**_r_* = 455.17	Mo *K*α radiation, λ = 0.71073 Å
Hexagonal, *R*3	Cell parameters from 2130 reflections
Hall symbol: -R 3	θ = 2.5–24.5°
*a* = 18.675 (2) Å	µ = 1.24 mm^−^^1^
*c* = 27.595 (3) Å	*T* = 298 K
*V* = 8334.6 (16) Å^3^	Block, red
*Z* = 18	0.32 × 0.30 × 0.27 mm
*F*(000) = 4176	

### Data collection

**Table:**

Bruker APEXII CCD area-detector diffractometer	4045 independent reflections
Radiation source: fine-focus sealed tube	2390 reflections with *I* > 2σ(*I*)
graphite	*R*_int_ = 0.126
ω scans	θ_max_ = 27.0°, θ_min_ = 2.2°
Absorption correction: multi-scan (*SADABS*; Sheldrick, 2004)	*h* = −19→22
*T*_min_ = 0.692, *T*_max_ = 0.730	*k* = −23→23
13818 measured reflections	*l* = −35→23

### Refinement

**Table:**

Refinement on *F*^2^	Primary atom site location: structure-invariant direct methods
Least-squares matrix: full	Secondary atom site location: difference Fourier map
*R*[*F*^2^ > 2σ(*F*^2^)] = 0.049	Hydrogen site location: inferred from neighbouring sites
*wR*(*F*^2^) = 0.135	H atoms treated by a mixture of independent and constrained refinement
*S* = 1.00	*w* = 1/[σ^2^(*F*_o_^2^) + (0.0437*P*)^2^] where *P* = (*F*_o_^2^ + 2*F*_c_^2^)/3
4045 reflections	(Δ/σ)_max_ = 0.001
257 parameters	Δρ_max_ = 0.40 e Å^−^^3^
9 restraints	Δρ_min_ = −0.44 e Å^−^^3^

### Special details

**Table:**

Geometry. All e.s.d.'s (except the e.s.d. in the dihedral angle between two l.s. planes) are estimated using the full covariance matrix. The cell e.s.d.'s are taken into account individually in the estimation of e.s.d.'s in distances, angles and torsion angles; correlations between e.s.d.'s in cell parameters are only used when they are defined by crystal symmetry. An approximate (isotropic) treatment of cell e.s.d.'s is used for estimating e.s.d.'s involving l.s. planes.
Refinement. Refinement of *F*^2^ against ALL reflections. The weighted *R*-factor *wR* and goodness of fit *S* are based on *F*^2^, conventional *R*-factors *R* are based on *F*, with *F* set to zero for negative *F*^2^. The threshold expression of *F*^2^ > σ(*F*^2^) is used only for calculating *R*-factors(gt) *etc*. and is not relevant to the choice of reflections for refinement. *R*-factors based on *F*^2^ are statistically about twice as large as those based on *F*, and *R*- factors based on ALL data will be even larger.

### Fractional atomic coordinates and isotropic or equivalent isotropic displacement parameters (Å^2^)

**Table:**

	*x*	*y*	*z*	*U*_iso_*/*U*_eq_	Occ. (<1)
Co1	0.55643 (3)	0.59070 (3)	0.127819 (16)	0.04116 (18)	
Cl1	0.98997 (7)	0.87669 (9)	0.09447 (5)	0.0861 (4)	
Cl2	0.41025 (9)	0.19824 (7)	0.01276 (5)	0.0928 (5)	
N1	0.63370 (19)	0.63087 (18)	0.17903 (10)	0.0430 (7)	
N2	0.47432 (18)	0.5496 (2)	0.07872 (10)	0.0453 (8)	
O1	0.63160 (15)	0.65983 (16)	0.08074 (9)	0.0539 (7)	
O2	0.47877 (16)	0.51745 (17)	0.17488 (9)	0.0503 (7)	
O3	0.58854 (15)	0.51130 (16)	0.11779 (8)	0.0514 (7)	
C1	0.7546 (2)	0.7142 (2)	0.12998 (13)	0.0433 (9)	
C2	0.7114 (2)	0.7059 (2)	0.08620 (13)	0.0455 (9)	
C3	0.7592 (2)	0.7521 (3)	0.04638 (14)	0.0538 (10)	
H3	0.7327	0.7476	0.0171	0.065*	
C4	0.8429 (2)	0.8033 (2)	0.04895 (15)	0.0536 (10)	
H4	0.8723	0.8338	0.0220	0.064*	
C5	0.8833 (2)	0.8092 (2)	0.09172 (16)	0.0540 (11)	
C6	0.8408 (2)	0.7664 (2)	0.13157 (14)	0.0505 (10)	
H6	0.8691	0.7716	0.1603	0.061*	
C7	0.7118 (2)	0.6768 (2)	0.17474 (13)	0.0436 (9)	
H7	0.7437	0.6871	0.2025	0.052*	
C8	0.5941 (3)	0.5974 (2)	0.22574 (12)	0.0543 (10)	
H8A	0.5756	0.6328	0.2402	0.065*	
H8B	0.6326	0.5939	0.2478	0.065*	
C9	0.5219 (3)	0.5132 (3)	0.21629 (14)	0.0587 (11)	
H9A	0.5410	0.4742	0.2106	0.070*	
H9B	0.4854	0.4945	0.2442	0.070*	
C10	0.4806 (2)	0.4271 (2)	0.06097 (12)	0.0459 (9)	
C11	0.5442 (2)	0.4424 (2)	0.09464 (13)	0.0446 (9)	
C12	0.5605 (3)	0.3772 (3)	0.10272 (14)	0.0549 (11)	
H12	0.5999	0.3841	0.1257	0.066*	
C13	0.5200 (3)	0.3042 (3)	0.07785 (15)	0.0608 (11)	
H13	0.5324	0.2626	0.0838	0.073*	
C14	0.4608 (3)	0.2926 (2)	0.04396 (14)	0.0575 (11)	
C15	0.4415 (2)	0.3523 (2)	0.03509 (13)	0.0518 (10)	
H15	0.4022	0.3438	0.0117	0.062*	
C16	0.4515 (2)	0.4846 (2)	0.05371 (13)	0.0458 (9)	
H16	0.4134	0.4735	0.0290	0.055*	
O4	0.52234 (16)	0.67011 (15)	0.14056 (9)	0.0524 (7)	0.638 (18)
C17	0.4410 (3)	0.6051 (3)	0.07147 (16)	0.0722 (14)	0.638 (18)
H17A	0.3836	0.5734	0.0615	0.087*	0.638 (18)
H17B	0.4717	0.6446	0.0460	0.087*	0.638 (18)
C18	0.4473 (6)	0.6495 (6)	0.1169 (4)	0.061 (3)	0.638 (18)
H18A	0.4455	0.6994	0.1098	0.073*	0.638 (18)
H18B	0.4010	0.6148	0.1378	0.073*	0.638 (18)
O4'	0.52234 (16)	0.67011 (15)	0.14056 (9)	0.0524 (7)	0.362 (18)
C17'	0.4410 (3)	0.6051 (3)	0.07147 (16)	0.0722 (14)	0.362 (18)
H17C	0.4407	0.6163	0.0372	0.087*	0.362 (18)
H17D	0.3844	0.5787	0.0831	0.087*	0.362 (18)
C18'	0.4876 (12)	0.6776 (8)	0.0957 (6)	0.062 (5)	0.362 (18)
H18C	0.4536	0.7022	0.1024	0.074*	0.362 (18)
H18D	0.5324	0.7153	0.0747	0.074*	0.362 (18)
H2	0.4293 (12)	0.507 (3)	0.1794 (16)	0.080*	

### Atomic displacement parameters (Å^2^)

**Table:**

	*U*^11^	*U*^22^	*U*^33^	*U*^12^	*U*^13^	*U*^23^
Co1	0.0373 (3)	0.0420 (3)	0.0389 (3)	0.0159 (3)	−0.0011 (2)	−0.0015 (2)
Cl1	0.0381 (7)	0.0883 (10)	0.1121 (10)	0.0166 (6)	0.0011 (6)	0.0099 (8)
Cl2	0.1280 (12)	0.0464 (7)	0.0801 (8)	0.0257 (7)	−0.0243 (8)	−0.0136 (6)
N1	0.046 (2)	0.0418 (18)	0.0354 (16)	0.0175 (16)	−0.0015 (14)	−0.0045 (13)
N2	0.0376 (18)	0.055 (2)	0.0399 (17)	0.0202 (16)	−0.0004 (13)	−0.0005 (15)
O1	0.0357 (16)	0.0643 (18)	0.0447 (14)	0.0121 (14)	−0.0010 (11)	0.0086 (13)
O2	0.0366 (15)	0.0582 (17)	0.0497 (15)	0.0188 (14)	0.0006 (12)	0.0096 (12)
O3	0.0427 (16)	0.0563 (18)	0.0551 (15)	0.0246 (14)	−0.0131 (12)	−0.0166 (13)
C1	0.043 (2)	0.040 (2)	0.046 (2)	0.0194 (19)	−0.0029 (17)	−0.0037 (16)
C2	0.039 (2)	0.048 (2)	0.048 (2)	0.021 (2)	0.0018 (17)	−0.0018 (17)
C3	0.047 (3)	0.062 (3)	0.049 (2)	0.025 (2)	0.0038 (18)	0.0041 (19)
C4	0.040 (2)	0.054 (3)	0.062 (3)	0.019 (2)	0.0103 (19)	0.009 (2)
C5	0.033 (2)	0.047 (2)	0.077 (3)	0.017 (2)	0.004 (2)	0.000 (2)
C6	0.044 (2)	0.055 (3)	0.058 (2)	0.029 (2)	−0.0064 (19)	−0.009 (2)
C7	0.047 (3)	0.043 (2)	0.042 (2)	0.024 (2)	−0.0092 (17)	−0.0067 (16)
C8	0.062 (3)	0.052 (3)	0.035 (2)	0.018 (2)	0.0011 (18)	−0.0012 (17)
C9	0.055 (3)	0.065 (3)	0.048 (2)	0.024 (2)	−0.0023 (19)	0.011 (2)
C10	0.045 (2)	0.047 (2)	0.036 (2)	0.016 (2)	0.0040 (17)	0.0027 (17)
C11	0.039 (2)	0.048 (2)	0.041 (2)	0.0180 (19)	0.0028 (16)	−0.0037 (17)
C12	0.056 (3)	0.061 (3)	0.050 (2)	0.032 (2)	−0.0057 (19)	−0.005 (2)
C13	0.075 (3)	0.055 (3)	0.057 (3)	0.036 (3)	0.003 (2)	0.002 (2)
C14	0.072 (3)	0.044 (2)	0.045 (2)	0.020 (2)	0.004 (2)	0.0004 (18)
C15	0.051 (3)	0.045 (2)	0.043 (2)	0.012 (2)	−0.0058 (17)	−0.0031 (18)
C16	0.034 (2)	0.052 (3)	0.0365 (19)	0.0104 (19)	0.0005 (15)	0.0010 (18)
O4	0.0542 (17)	0.0457 (17)	0.0576 (16)	0.0252 (14)	−0.0065 (13)	−0.0045 (12)
C17	0.080 (3)	0.102 (4)	0.065 (3)	0.067 (3)	−0.009 (2)	−0.008 (3)
C18	0.047 (5)	0.064 (6)	0.080 (6)	0.034 (5)	−0.002 (4)	0.003 (4)
O4'	0.0542 (17)	0.0457 (17)	0.0576 (16)	0.0252 (14)	−0.0065 (13)	−0.0045 (12)
C17'	0.080 (3)	0.102 (4)	0.065 (3)	0.067 (3)	−0.009 (2)	−0.008 (3)
C18'	0.063 (9)	0.051 (7)	0.073 (8)	0.030 (6)	−0.006 (6)	−0.001 (6)

### Geometric parameters (Å, °)

**Table:**

Co1—O1	1.874 (2)	C7—H7	0.9300
Co1—O3	1.877 (3)	C8—C9	1.496 (5)
Co1—N1	1.887 (3)	C8—H8A	0.9700
Co1—N2	1.897 (3)	C8—H8B	0.9700
Co1—O4	1.916 (3)	C9—H9A	0.9700
Co1—O2	1.918 (3)	C9—H9B	0.9700
Cl1—C5	1.747 (4)	C10—C15	1.405 (5)
Cl2—C14	1.753 (4)	C10—C11	1.420 (5)
N1—C7	1.275 (4)	C10—C16	1.440 (5)
N1—C8	1.462 (4)	C11—C12	1.413 (5)
N2—C16	1.270 (4)	C12—C13	1.367 (5)
N2—C17	1.465 (5)	C12—H12	0.9300
O1—C2	1.305 (4)	C13—C14	1.380 (6)
O2—C9	1.423 (4)	C13—H13	0.9300
O2—H2	0.855 (10)	C14—C15	1.356 (6)
O3—C11	1.298 (4)	C15—H15	0.9300
C1—C6	1.404 (5)	C16—H16	0.9300
C1—C2	1.418 (5)	O4—C18	1.414 (7)
C1—C7	1.447 (5)	C17—C18	1.474 (8)
C2—C3	1.406 (5)	C17—H17A	0.9700
C3—C4	1.368 (5)	C17—H17B	0.9700
C3—H3	0.9300	C18—H18A	0.9700
C4—C5	1.375 (5)	C18—H18B	0.9700
C4—H4	0.9300	C18'—H18C	0.9700
C5—C6	1.359 (5)	C18'—H18D	0.9700
C6—H6	0.9300		
			
O1—Co1—O3	90.97 (12)	N1—C8—C9	107.0 (3)
O1—Co1—N1	94.87 (12)	N1—C8—H8A	110.3
O3—Co1—N1	86.66 (12)	C9—C8—H8A	110.3
O1—Co1—N2	87.65 (12)	N1—C8—H8B	110.3
O3—Co1—N2	95.06 (12)	C9—C8—H8B	110.3
N1—Co1—N2	176.94 (12)	H8A—C8—H8B	108.6
O1—Co1—O4	91.05 (11)	O2—C9—C8	108.6 (3)
O3—Co1—O4	177.77 (11)	O2—C9—H9A	110.0
N1—Co1—O4	92.22 (12)	C8—C9—H9A	110.0
N2—Co1—O4	85.98 (12)	O2—C9—H9B	110.0
O1—Co1—O2	178.46 (12)	C8—C9—H9B	110.0
O3—Co1—O2	87.87 (12)	H9A—C9—H9B	108.4
N1—Co1—O2	86.08 (12)	C15—C10—C11	119.9 (4)
N2—Co1—O2	91.44 (12)	C15—C10—C16	118.0 (4)
O4—Co1—O2	90.13 (12)	C11—C10—C16	122.0 (3)
C7—N1—C8	122.3 (3)	O3—C11—C12	118.1 (3)
C7—N1—Co1	126.0 (2)	O3—C11—C10	125.2 (4)
C8—N1—Co1	111.5 (2)	C12—C11—C10	116.7 (3)
C16—N2—C17	122.9 (3)	C13—C12—C11	122.0 (4)
C16—N2—Co1	126.3 (3)	C13—C12—H12	119.0
C17—N2—Co1	110.8 (3)	C11—C12—H12	119.0
C2—O1—Co1	126.1 (2)	C12—C13—C14	119.9 (4)
C9—O2—Co1	109.7 (2)	C12—C13—H13	120.1
C9—O2—H2	117 (3)	C14—C13—H13	120.1
Co1—O2—H2	127 (3)	C15—C14—C13	120.9 (4)
C11—O3—Co1	123.9 (2)	C15—C14—Cl2	120.5 (3)
C6—C1—C2	119.6 (3)	C13—C14—Cl2	118.6 (4)
C6—C1—C7	118.2 (3)	C14—C15—C10	120.5 (4)
C2—C1—C7	121.8 (3)	C14—C15—H15	119.8
O1—C2—C3	118.6 (3)	C10—C15—H15	119.8
O1—C2—C1	124.8 (3)	N2—C16—C10	124.5 (3)
C3—C2—C1	116.6 (3)	N2—C16—H16	117.8
C4—C3—C2	122.7 (4)	C10—C16—H16	117.8
C4—C3—H3	118.6	C18—O4—Co1	111.8 (3)
C2—C3—H3	118.6	N2—C17—C18	109.8 (4)
C3—C4—C5	119.4 (4)	N2—C17—H17A	109.7
C3—C4—H4	120.3	C18—C17—H17A	109.7
C5—C4—H4	120.3	N2—C17—H17B	109.7
C6—C5—C4	120.8 (4)	C18—C17—H17B	109.7
C6—C5—Cl1	120.6 (3)	H17A—C17—H17B	108.2
C4—C5—Cl1	118.5 (3)	O4—C18—C17	109.6 (5)
C5—C6—C1	120.9 (4)	O4—C18—H18A	109.7
C5—C6—H6	119.6	C17—C18—H18A	109.7
C1—C6—H6	119.6	O4—C18—H18B	109.7
N1—C7—C1	125.1 (3)	C17—C18—H18B	109.7
N1—C7—H7	117.5	H18A—C18—H18B	108.2
C1—C7—H7	117.5	H18C—C18'—H18D	107.5

### Hydrogen-bond geometry (Å, °)

**Table:**

*D*—H···*A*	*D*—H	H···*A*	*D*···*A*	*D*—H···*A*
O2—H2···O4^i^	0.86 (1)	1.59 (1)	2.436 (4)	173 (5)

Symmetry codes: (i) *y*−1/3, −*x*+*y*+1/3, −*z*+1/3.
